# miR-27b-3p a Negative Regulator of DSB-DNA Repair

**DOI:** 10.3390/genes12091333

**Published:** 2021-08-27

**Authors:** Ricardo I. Peraza-Vega, Mahara Valverde, Emilio Rojas

**Affiliations:** Departamento de Medicina Genómica y Toxicología Ambiental, Instituto de Investigaciones Biomédicas, Universidad Nacional Autónoma de México, Ciudad Universitaria, Ciudad de México 04510, Mexico; ricardoivan@gmail.com (R.I.P.-V.); mahara@iibiomedicas.unam.mx (M.V.)

**Keywords:** DNA repair, miR-27b-3p, gene regulation, comet assay, double-strand break, cancer

## Abstract

Understanding the regulation of DNA repair mechanisms is of utmost importance to identify altered cellular processes that lead to diseases such as cancer through genomic instability. In this sense, miRNAs have shown a crucial role. Specifically, miR-27b-3 biogenesis has been shown to be induced in response to DNA damage, suggesting that this microRNA has a role in DNA repair. In this work, we show that the overexpression of miR-27b-3p reduces the ability of cells to repair DNA lesions, mainly double-stranded breaks (DSB), and causes the deregulation of genes involved in homologous recombination repair (HRR), base excision repair (BER), and the cell cycle. DNA damage was induced in BALB/c-3T3 cells, which overexpress miR-27b-3p, using xenobiotic agents with specific mechanisms of action that challenge different repair mechanisms to determine their reparative capacity. In addition, we evaluated the expression of 84 DNA damage signaling and repair genes and performed pathway enrichment analysis to identify altered cellular processes. Taken together, our results indicate that miR-27b-3p acts as a negative regulator of DNA repair when overexpressed.

## 1. Introduction

Maintaining genomic integrity is crucial for the proper functioning of cells and the faithful transmission of genetic information to new cells. There are numerous extrinsic and intrinsic factors that can cause various types of alterations in the structure of DNA; therefore, cells have developed different mechanisms responsible for repairing a variety of DNA lesions. Malfunction of DNA damage signaling and repair mechanisms promotes accumulation of mutations, genomic instability, and, consequently, aging, neurodegeneration, immunodeficiency, cancer, and cell death [1,2].

There are several mechanisms employed by cells to deal with different types of DNA lesions. Among them, base excision repair (BER) permits the repair of lesions that do not greatly distort the structure of the DNA molecule, such as oxidative lesions caused by the interaction of DNA with reactive oxygen species (ROS) and base deamination or depurination caused by spontaneous hydrolysis [3,4,5]; nucleotide excision repair (NER) is responsible for repairing bulky chemical adducts and interstrand crosslinks that significantly distort the structure of DNA, including pyrimidine–pyrimidone (6-4) photoproducts (6-4PPs) and cyclobutane pyrimidine dimers (CPDs) caused by ultraviolet (UV) light [6,7]. On the other hand, nonhomologous end joining (NHEJ) and homologous recombination repair (HRR) are responsible for repairing double-strand breaks (DSBs), which are considered the most cytotoxic type of lesion occurring on DNA due to its mutagenic effects [8,9]. Additionally, cells have mechanisms to temporarily tolerate DNA damage (DDT), which differs conceptually from DNA repair since instead of restoring DNA to its proper sequence and structure, the injury remains in the DNA. There are two distinct types of DDT: one is template-switching (TS), in which the stalled nascent strand temporarily changes to the newly synthesized undamaged sister strand for replication on the lesion; the other is translesion synthesis (TLS), in which the replicative DNA polymerase is temporarily replaced by a special TLS polymerase that can replicate through DNA lesions, contributing to gains in mutagenesis and chemoresistance [10].

For the correct signaling and repair of DNA damage, strict regulation of gene expression associated with these processes is necessary. In this sense, microRNAs (miRNAs) (~22 nt) act as post-transcriptional modulators of gene expression, causing the repression or degradation of mRNA through imperfect or perfect base pairing, thus modulating many cellular processes [11]. There is ample evidence that miRNAs regulate the expression of DNA repair genes [12,13,14,15]. Therefore, alterations in the expression of these miRNAs can alter the DNA repair capacity, thus compromising genomic maintenance, an enabling characteristic for tumor progression [16]. Furthermore, changes in miRNA expression have also been associated with sensitivity to cancer treatment [17,18,19]. These findings have opened a field of interest regarding miRNAs as key actors to improve cancer diagnosis and therapy strategies [18,20,21,22]. However, more studies are required to understand the specific role of many miRNAs in DNA repair, tumor development, and sensitivity to therapy.

Among the miRNAs associated with cancer, it has been observed that the expression of miR-27b-3p is frequently deregulated in several types of human tumors. miR-27b-3p has been reported to be overexpressed in cervical, breast, and glioma cancer [23,24,25] and downregulated in prostate, colorectal, gastric, bladder, and lung cancer [26,27,28,29,30]. Furthermore, miR-27b-3p biogenesis has been shown to be induced in response to DNA damage [31]. However, there are no studies that explain the role of miR-27b-3p in regulating DNA repair. In this study, we show that miR-27b-3p negatively regulates DNA damage repair. The overexpression of this miRNA in BALB/c-3T3 cells treated with different xenobiotic agents (ionizing radiation, ferric chloride, ultraviolet light, and doxorubicin), with known mechanisms of action, resulted in a significant reduction in DNA repair capacity in cultures treated with doxorubicin, which correlates with changes in the expression of genes mainly involved in the repair of double-stranded breaks through HRR. However, we did not find an effect on DNA repair when we inhibited this microRNA. The combination of these findings indicates that miR-27b-3p acts as a negative regulator of DNA repair.

## 2. Materials and Methods

### 2.1. Cell Line and Culture

Mouse embryonic fibroblasts from the BALB/c-3T3 A31-1-1 cell line were acquired from the American Type Culture Collection (ATCC). The cell line was cryopreserved in liquid nitrogen in Dulbecco’s Modified Eagle’s Medium (DMEM) (ThermoFisher Scientific, Waltham, MA, USA) supplemented with 5% DMSO until use. For cell culture, cells were seeded at a density of 1 × 10^5^ on 10 mm plastic culture dishes in DMEM supplemented with 10% fetal calf serum (FCS) and 1% antibiotic–antimycotic (ThermoFisher Scientific) and maintained at 37 °C in a humidified atmosphere with 5% CO_2_.

### 2.2. Cell Transfection

In order to induce changes in miR-27b-3p expression, BALB/c-3T3 cells were transfected with an hsa-miR-27b-3p mimic and hsa-miR-27b-3p inhibitor nucleotide sequences (Ambion, Austin, TX, USA). siPORT NeoFX Transfection Agent (Invitrogen, Waltham, MA, USA) was applied according to the manufacturer’s protocol. Cells transfected with an empty transfection agent were used as control. To perform cell transfection, 2.4 × 10^5^ cells were used per tested condition.

### 2.3. Treatment with DNA Damaging Agents

In order to evaluate if the overexpression of miR-27b alters the ability of cells to repair DNA damage, cells were exposed to ultraviolet light (UV), FeCl_3_, ionizing radiation (IR), and doxorubicin hydrochloride (DOX). These DNA damaging agents induce different types of DNA lesions that are repaired through different DNA repair mechanisms (Table 1). Control cells (containing only siPORT) and cells transfected with the miR-27b-3p mimic were exposed to UV, FeCl_3_, IR, and DOX. Cells transfected with the miR-27b-3p inhibitor were exposed to IR and DOX. DOX, as an intercalator into DNA, inhibits type II topoisomerase-mediated DNA repair, thus preventing the ligation of DSBs [32,33]. Furthermore, DOX is oxidized to semiquinone, which is converted back to DOX in a process that produces ROS [34,35], which can interact with DNA and other macromolecules to produce oxidative lesions [32]. Infrared radiation causes the DNA strand to break, depending on its energy, in addition to the loss of electrons in biomolecular bonds, thus altering its structure and function. IR can also produce ROS when high-energy particles interact with water molecules within the cell; therefore, IR generates DSB, SSB, and oxidative DNA damage [36,37]. Iron can promote the generation of ROS, particularly hydroxyl radicals (• OH), through the Fenton reaction, which, in turn, promotes the production of oxidative lesions in DNA [38]. Ultraviolet light produces cyclobutane–pyrimidine dimers (CPD) and 6-4 photoproducts (6-4PP) in DNA [39].

At 24 h after transfection, the cell culture medium was replaced with fresh medium, and cells were exposed to different xenobiotic agents: UV light employing a Hoefer UVC-500 crosslinker; FeCl_3_ for 1 h; 7.5 Gy of IR using a Gammacell-1000 irradiator with a Cesium-137 radioactive source; DOX for 1 h. The doses and concentrations for each DNA damaging agent were previously determined by performing survival and DNA damage curves (Table S1). We selected the doses and concentrations that allowed us to generate significantly higher levels of DNA damage than the basal level of untreated cells (*p* < 0.05, Student’s *t*-tests).

### 2.4. DNA Repair Measurement through Comet Assay

DNA damage levels were evaluated at three different post-treatment time intervals through comet assay. This allowed us to compare DNA damage values from each interval and determine the DNA repair capacity between transfected and control conditions. For UV, FeCl_3_, and IR, post-treatment intervals were 0 (induced DNA damage), 1, and 24 h after treatments. Since DOX inhibits type II topoisomerases, DSBs become evident after DNA replication. Therefore, for DOX treatment, DNA damage measurement time intervals were 24 (induced DNA damage), 36, and 48 h after treatment.

At the end of each post-treatment interval, the cells were centrifuged at 250× *g* for 5 min, resuspended with 0.5% low melting point (LMP) agarose and placed on 76 × 26 mm glass slides previously coated with 0.5% regular agarose, and covered with a 24 × 50 mm coverslip. Slides were stored at 4 °C for 5 min to allow LMP solidification. Coverslips were carefully removed, and an additional layer of cell-free 0.5% LMP was placed on top of the previous layer, covered with coverslips, and stored again at 4 °C for 5 min. Coverslips were removed, and the slides were submerged in lysing solution (2.5 M NaCl, 100 mM EDTA disodium, 10 mM Tris-Base, pH 10, supplemented in fresh with 10% DMSO and 1% Triton X-100) at 4 °C for 24 h. After lysis, slides were placed on an electrophoresis chamber, placed on ice containing alkaline (pH > 13) (300 mM NaOH, 1 mM EDTA disodium) or neutral (pH = 8.5) (90 mM boric acid, 5 mM EDTA disodium, 117 mM Tris-Base) buffer and left for DNA unwinding for 20 or 45 min, respectively. After unwinding, electrophoresis was conducted at 300 mA/25 V (0.8 V/cm) for 20 min in the alkaline version or 20 mA/25 V (0.05 V/cm) for 2 h in the neutral version. Once electrophoresis was finished, the slides were rinsed with a neutralization buffer (pH = 7.5, 0.4 M Tris-Base) and dehydrated with 96% ethanol. The slides were stained with 2 µg/mL ethidium bromide and examined at 20X magnification with an Olympus BX60 fluorescence microscope connected to a CCD camera for image visualization in Komet v5.0 software for DNA damage evaluation. Olive tail moment (OTM) [40] values from 100 comets per condition were scored at the different post-treatment intervals. Three experimental replicates were made. The OTM values of transfected and non-transfected cells were compared using ANOVA and Dunnet’s multiple comparison tests (*p* < 0.05).

By comparing the OTM values of the alkaline and neutral comet assays, it is possible to detect and differentiate between DNA single- and double-strand breaks. The comet neutral assay allows the detection of DNA damage caused mainly by double-strand breaks (DSB), while the alkaline comet assay allows the detection of DSB, SSB, as well as alkali–labile sites such as apurinic and apyrimidinic sites (APs), which become the strand breaks in the presence of alkaline pH [41,42].

### 2.5. Total RNA Isolation

The LEV simplyRNA Cells kit (Promega, Madison, WI, USA) was used to extract total RNA from transfected and control cells according to the manufacturer’s protocol using an automated Maxwell 16 instrument (Promega). Total RNA was extracted at 24 h after miR-27b mimic and inhibitor transfection to evaluate miR-27b and DNA repair gene expression. Total RNA was also extracted at 48 h after doxorubicin treatment to evaluate DNA repair gene expression during the repair of doxorubicin-induced lesions. Genomic DNA was removed from samples using the DNase I included in the kit’s prefilled cartridges. RNA was quantified using a Multiskan GO instrument (ThermoFisher). The purity of isolated RNA was evaluated by measuring the 260/280 optical density ratio, which ranged from 1.9 to 2.2 for all samples.

### 2.6. Reverse Transcription and Quantitative PCR to Evaluate miR-27b Expression

Measurement of miR-27b-3p expression at 24 h after mimic and inhibitor transfection was conducted by RT-qPCR using the TaqMan MicroRNA Reverse Transcription and MicroRNA Assays kit (Applied Biosystems, Waltham, MA, USA) containing specific hsa-miR-27b-3p primers according to manufacturer’s instructions. U6 small nuclear RNA (*Rnu6*) was used as endogenous control to normalize miRNA expression. Each sample was analyzed in triplicate in 15 µL reactions. The relative expression of miR-27b in transfected and non-transfected cells was analyzed by the comparative Ct method [43].

### 2.7. Reverse Transcription and Quantitative PCR for Gene Expression Profiling

To determine the expression of DNA repair genes in miR-27b mimic and inhibitor transfected cells and control cells, cDNA was synthesized using the RT2 First Strand kit (Qiagen, Hilden, Germany) following the manufacturer’s instructions. Aliquots with 0.5 µg of total RNA from each sample were used for cDNA synthesis. The expression of 84 DNA repair genes was analyzed by quantitative PCR using the 96-well DNA Damage Signaling Pathway RT2 Profiler PCR array (PAMM-029ZF, Qiagen) according to the manufacturer’s protocol in a LightCycler 96 Real-Time PCR system (Roche Life Science). The genes included in the PCR array are: *Abl1, Apex1, Atm, Atr, Atrx, Bax, Blm, Brca1, Brca2, Brip1, Cdc25a, Cdc25c, Cdkn1a, Chek1, Chek2, Dclre1a, Ddb2, Ddit3, Ercc1, Ercc2, Exo1, Fanca, Fancc, Fancd2, Fancg, Fen1, Gadd45a, Gadd45g, H2afx, Hus1, Lig1, Mbd4, Mcph1, Mdc1, Mgmt, Mif, Mlh1, Mlh3, Mpg, Mre11a, Msh2, Msh3, Nbn, Nthl1, Ogg1, Parp1, Parp2, Pcna, Pms2, Pole, Polh, Poli, Ppm1d, Ppp1r15a, Prkdc, Pttg1, Rad1, Rad17, Rad18, Rad21, Rad50, Rad51, Rad51c, Rad51l1, Rad52, Rad9, Rev1, Rnf8, Rpa1, Smc1a, Smc3, Sumo1, Terf1, Topbp1, Trp53, Trp53bp1, Ung, Wrn, Xpa, Xpc, Xrcc1, Xrcc2, Xrcc3,* and *Xrcc6.* PCR array data analysis was performed by comparative Ct method using Qiagen’s Data Analysis Center. The expression data were normalized to five endogenous control genes (*Actb, B2m, Gapdh, Gusb,* and *Hsp90ab1*) included in the PCR array.

### 2.8. miRNA Target Prediction and Pathway Enrichment Analysis

A list of genes from *Mus musculus*, annotated to the Gene Ontology Term “DNA Repair”, was obtained from the AmiGO web database [44]. The DNA Repair gene list was used as a query search on the miRWalk 2.0 web platform [45] to obtain predicted miR-27b-3p–gene interactions using the following parameters: predicted interactions on CDS, 5′ or 3′ UTRs, minimum *p*-value <0.05, 7 nt as minimum seed length, and including all available databases (miRWalk, miRDB, PITA, MicroT4, miRMap, RNA22, miRanda, miRNAMap, RNAhybrid, miRBridge, PICTAR2, and TargetScan). Only predicted genes that appeared in at least half of the specified databases were considered for this study.

Deregulated genes in miR-27b-3p-overexpressing cells were submitted to Reactome pathway enrichment analysis [46]. This pathway enrichment analysis allowed us to determine the functional pathways representative of the deregulated genes.

## 3. Results

### 3.1. miR-27b-3p Overexpression and DNA Repair Capacity

**Figure 1:**
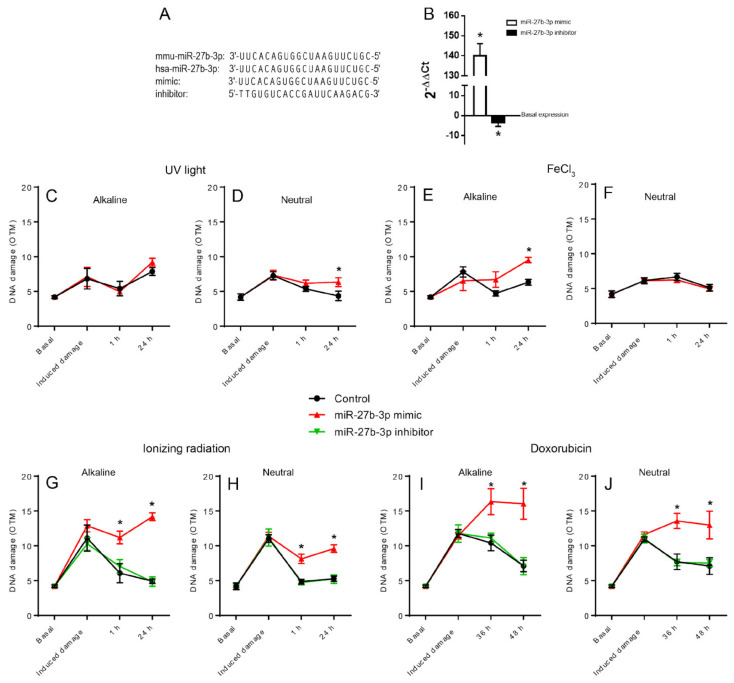
Overexpression of miR-27b-3p reduces DNA repair capacity. (**A**) Alignment of human and murine miR-27b-3p sequences; mimic and inhibitor sequences employed. (**B**) Quantification of miR-27b-3p expression in mimic and inhibitor transfected in BALB/c-3T3 cells. The data of each experimental condition were normalized with respect to the basal expression of Rnu6. The normalized data are expressed with respect to the control and presented as mean ± SD from three replicas using the Ct comparative method and Student’s *t*-test [41]. (**C**–**J**) Measurement of DNA damage induced by four DNA damaging agents at three post-treatment time intervals in cells transfected with miR-27b-3p mimic, inhibitor, and control through alkaline and neutral comet assays. (**C**,**D**) UV light (126 mJ); (**E**,**F**) ferric chloride (50 µM/1 h); (**G**,**H**) ionizing radiation (7.5 Gy); (**I**,**J**) doxorubicin (50 µM/1 h). Representative comet images are included in Figure S1. DNA damage is represented as Olive Tail Moment (OTM) [40]. OTM values are presented as mean ± SD from 3 independent experiments. (*) Statistically different from the control condition in the same interval using ANOVA followed by Dunn’s multiple comparison test (*p* < 0.05).

To determine whether the overexpression of miR-27b-3p affected the cells’ ability to repair DNA damage, we induced overexpression of miR-27b-3p by transfecting BALB/c-3T3 cells with the mimic hsa-miR-27b-3p (Figure 1A,B). The transfected and control cells were then exposed to four different agents that damage DNA: ultraviolet (UV) light, ferric chloride (FeCl_3_), doxorubicin (DOX), and ionizing radiation (IR). DNA damage levels were measured in both alkaline and neutral comet assays at three different post-treatment intervals to compare the ability of cells to repair DNA damage induced by the agents employed. Table 1 shows a summary of the type of injuries induced by the agents used and the associated repair mechanisms.

The alkaline comet assay revealed that overexpression of miR-27b-3p mainly reduced the ability of cells to repair DNA damage caused by FeCl_3_, IR, and DOX (Figure 1C,E,G,I), while the results of the neutral version, which detects DSB, show how the overexpression of miRNA27b-3p inhibits the repair of cells treated with UV light, IR, and DOX (Figure 1D,H,J). However, we did not observe any change when cells were treated with FeCl_3_ (Figure 1F). These findings suggest that miR-27b-3p overexpression caused the downregulation of genes primarily involved in DSB repair, either by NHEJ or HRR, and the repair of oxidative injury by BER.

Furthermore, since the inhibition of this miRNA has been implicated in some types of human cancers, we explored whether the inhibition of miR-27b3p generated any effect on DNA repair capacity. Cells were transfected with a miR-27b-3p inhibitor to reduce miRNA expression (Figure 1A,B). These cells were exposed to DOX and IR, the treatments with which we saw the most notable effect of miR-27b-3p overexpression; we tested their ability to remove DNA damage using the comet assay. The downregulation of miR-27b-3p did not alter the ability of cells to repair DNA damage, as observed from the OTM values of cells transfected with the miR-27b-3p inhibitor compared to control cells (Figure 1G–J).

### 3.2. miR-27b-3p is Predicted to Modulate a High Number of DNA Repair Genes

**Figure 2:**
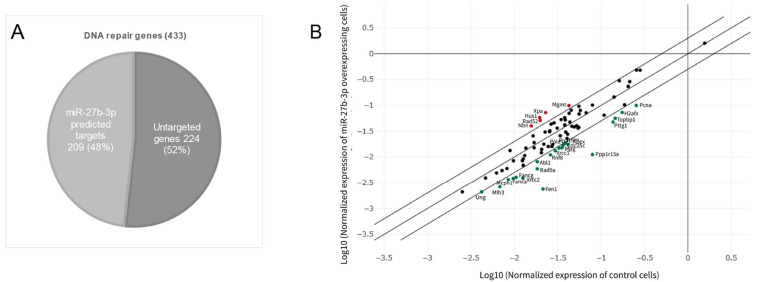
miR-27b-3p overexpression induces changes in DNA repair gene expression. (**A**) Proportion of DNA repair genes predicted to be regulated by miR-27b-3. (**B**) Gene expression pattern induced by miR-27b-3p overexpression. The scatter plot compares the normalized expression of every gene on the PCR array between the control and miR-27b-3p overexpressing cells by plotting them against one another to quickly visualize large gene expression changes. The center diagonal line indicates unchanged gene expression, while the outer diagonal lines indicate the selected fold regulation threshold. Fold-change (2^(-Delta Delta CT)) is the normalized gene expression (2^(- Delta CT)) in the test sample divided by the normalized gene expression (2^(- Delta CT)) in the control sample. Fold-change values greater than one indicate a positive or upregulation. Fold-change values less than one indicate a negative or downregulation. The *p*-values were calculated based on a Student’s *t*-test of the replicate 2^(- Delta CT) values for each gene in the control and miR-27b-3p overexpressing groups, and *p*-values less than 0.05 were considered for analysis. The *p*-value calculation used is based on parametric, unpaired, two-sample equal variance, two-tailed distribution—a method widely accepted in scientific literature. Upregulated genes: Nbn, Rad52, Hus1, Xpa, and Mgmt. Downregulated genes: *Ung, Mlh3, Mcpn1, Fanca, Fancg, Xrcc2, Fen1, Rad9a, Abl1, Rnf8, Xrcc1, Mpg, Cdc25c, Pole, Rpa1, Blm, Apex, Ppp1r15a, Pttg1, Topbp1, H2afx,* and *Pcna*.

As a first approach to evaluate if miR-27b-3p could modulate the expression of DNA repair genes, we performed an in silico analysis to determine the genes predicted to be targeted by miR-27b-3p. We obtained 209 putative miR-27b target genes classified within the gene ontology term “DNA Repair”, which represents 48% of the total genes classified within that term (Figure 2A). These findings suggested that the overexpression of miR-27b-3p could result in the repression of a large number of DNA repair genes, thus reducing the ability of cells to repair DNA damage, as observed in our model when miR-27b-3p was overexpressed.

### 3.3. miR-27b-3p Overexpression Alters the Expression of DNA Repair Genes

**Figure 3:**
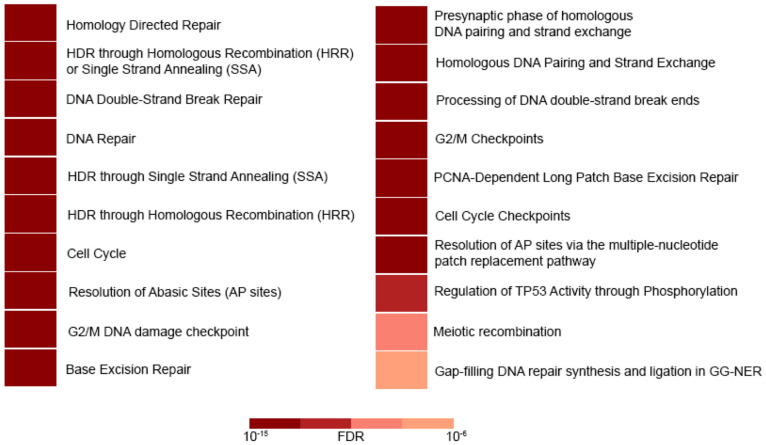
Reactome pathway enrichment analysis [45]. Deregulated genes in cells that overexpress miR-27b-3p act mainly in DSB repair through HRR and other closely related processes. Most of them enriched processes, including the 27 deregulated genes observed in the miR-27b-3p overexpressing cells. Color intensities are representatives of false discovery rate values (FDR).

To determine whether the reduction in DNA repair capacity was related to the DNA repair gene deregulation caused by miR-27b-3p overexpression, we analyzed the expression profile of the 84 genes included in the 96-well DNA Damage Signaling and Repair PCR array. Cells overexpressing miR-27b-3p showed downregulation of 22 genes and upregulation of 5 genes in comparison to control cells (2-fold) (Figure 2B). According to Reactome pathway enrichment analysis, the cell processes most represented by these 27 genes are related to DSB repair through homologous recombination, cell cycle regulation, and BER (Figure 3, Table 2 and Table S1).

### 3.4. Dysregulated DNA Repair in Doxorubicin-Damaged miR-27b-3p-Overexpressed Cells

**Figure 4:**
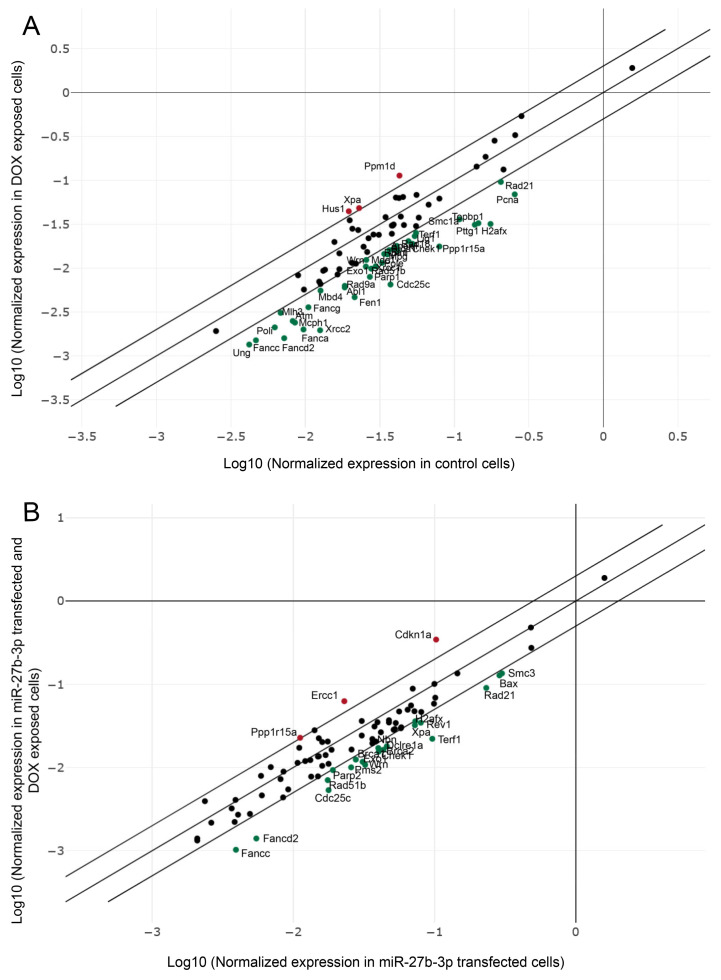
Gene expression pattern induced by doxorubicin. (**A**) Expression pattern of 84 repair genes induced by doxorubicin in control cells. (**B**) Expression pattern of 84 repair genes induced by doxorubicin in miR-27b-3p overexpressing cells. The scatter plot compares the normalized expression of every gene on the PCR array between the two selected groups by plotting them against one another to quickly visualize large gene expression changes. The center diagonal line indicates unchanged gene expression, while the outer diagonal lines indicate the selected fold regulation threshold. Fold-change (2^(-Delta Delta CT)) is the normalized gene expression (2^(- Delta CT)) in the test sample divided the normalized gene expression (2^(- Delta CT)) in the control sample. Fold-regulation represents fold-change results in a biologically meaningful way. Fold-change values greater than one indicate a positive or upregulation. Fold-change values less than one indicate a negative or downregulation. The *p*-values are calculated based on a Student’s *t*-test of the replicate 2^(- Delta CT) values for each gene in the control and treatment groups, and *p*-values less than 0.05 were considered for analysis. The *p*-value calculation used is based on parametric, unpaired, two-sample equal variance, two-tailed distribution—a method widely accepted in scientific literature. The expression of DNA repair genes changes in response to doxorubicin exposure. Normalized expression of 84 DNA repair genes at 48 h after doxorubicin treatment. Secondary lines represent a 2-fold threshold for upregulated (red) and downregulated (green) genes.

Despite the fact that miR-27b-3p overexpression negatively regulates many genes of various repair mechanisms (Figure 2B), we wanted to explore the expression pattern of the same 84 genes 48 h after doxorubicin treatment. We begin by analyzing the expression patterns of the DNA repair genes that showed functionality by DOX treatment (Figure 1I,J), and we show them in Figure 4A. The pattern shows three upregulated genes (*Hus, Ppm1d,* and *Xpa)* and 37 downregulated genes *(Abl1, Apex1, Atm, Bnm, Brca1, Cdc25c, Chek1, Exo1, Fanca, Fancc, Fanccd2, Fancg, Fen1, H2afx, lig1, Mbd4, Mcph1, Mdc1, Mlh3, Mpg, Parp1, Pcna, Pole, Poli, Ppp1r15a, Pttg1, Rad18, Rad21, Rad51c, Rad51b, Rad9a, Rpa1, Smc1a, Terf1, Topbp1, Ung,* and *Wrn*). In contrast, DOX-induced expression patterns under miR-27b-3p overexpression related to dysfunctional repair (Figure 1I,J) are shown in Figure 4B. Although the overall patterns are similar, the genes involved are different. The upregulated genes are *Ppp1r15a*, *Ercc1*, and *Cdkn1a* and the 20 downregulated genes are *Fancc, Fanccd2, Cdc25c, Rad51b, Parp, Pms2, Exo1, Brca1, Check1, Brca2, Dclre1a, Nbn, Xpa, Terf1, Xpa, Rev1, H2afx, Rad21, Bax,* and *Smc3*.

**Figure 5:**
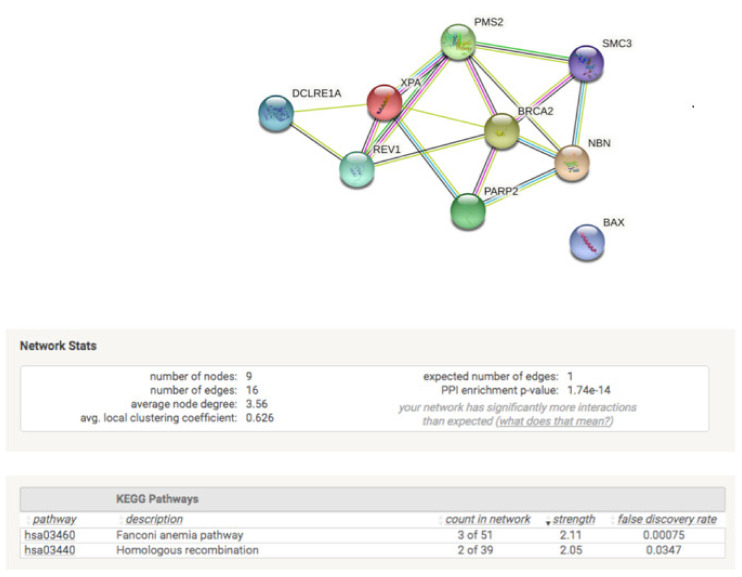
Protein interaction analysis. Differential study of protein interactions of DNA repair genes between cells that overexpress miR27b-3p treated with doxorubicin and genes that are not expressed in BALB/c-3T3 cells treated with doxorubicin. STRING analysis (v11.5) was performed using fold shift expression values [47].

Trying to understand how these genetic patterns are related to the loss of reparative functionality acquired by the overexpression of miR-27b-3p, we performed a protein interaction analysis (STRING) from the genes that differ between the patterns shown in Figure 4. The analysis by STRING (Figure 5) shows the robust interaction at the protein level of genes, which have more interactions than expected; the most impacted pathways are the Fanconi anemia pathway and homologous recombination. It is important to note that this analysis only reflects the interactions favored by treatment with doxorubicin as a genotoxic agent, and that is why the number of genes included is only nine. Likewise, it should be noted that this analysis has not yet been validated by quantifying the proteins involved.

## 4. Discussion

In this study, we show that the overexpression of miR-27b-3p negatively affects the ability of cells to repair DNA damage, particularly DSBs and oxidative lesions, such as those induced by DOX, IR, and FeCl_3_ exposure (Figure 1E–J). The oxidative damage generated by treatment with FeCl_3_ is only evident in the alkaline comet assay since the generation of AP sites is an initial step during BER [48] (Figure 1E,F). UV light does not produce DSB directly but can be generated as a result of transcription/replication blockade due to the presence of CPD [39,49] or due to inadequate repair of temporary SSBs that occur during CPD repair through NER or, alternatively, TLS. This could explain why only 24 h after UV exposure, when a large proportion of cells have probably replicated their genome, there is an increase in the presence of DSBs detected in the neutral comet assay. However, the most severe effect of miR-27b-3p overexpression on DNA repair was observed in cells treated with FeCl_3_, IR, and DOX (Figure 1E–J). Inhibition of miR-27b-3p expression did not alter DNA repair capacity (Figure 1C–J), indicating that miR-27b-3p’s effect on DNA repair depends on its overexpression. We also demonstrated that the expression of 27 genes was deregulated (2-fold change) in miR-27b-3p-overexpressing cells. Consistent with the DNA repair capacity results, deregulated genes were mainly involved in BER, DSB repair through HRR, and cell cycle regulation (Figure 3, Table 2), thus suggesting a strong link between a reduction in DNA repair capacity and miR-27b-3p overexpression.

Among the BER-associated genes that were downregulated due to miR-27b-3p overexpression were *Mpg, Ung, Apex1 (Ape1), Fen1, Xrcc1, Pcna,* and *Pole* (Table 2). These genes are involved in the detection of modified bases, strand cleavage, scaffolding, and DNA synthesis. The MPG and UNG proteins are monofunctional glycosylases responsible for removing modified bases such as 3meA, 7meG, 3meG, U, and 5-FU [50]. APEX1 is an apurinic/apyrimidic endonuclease that cleaves the phosphodiester bond to produce an SSB [51]. PCNA and POLE are proteins responsible for DNA synthesis from the 3’-OH left by APEX1 at the cleavage site in the long-patch variant of BER [52]. FEN1 is an endonuclease that participates in long-patch BER and is responsible for removing the “flap” overhang that remains after new DNA section synthesis [51]. XRCC1 has no enzymatic activity but functions as a scaffolding point for other BER components such as APEX1 and other glycosylases [53]. The HRR involved genes that were downregulated in miR-27b-3p-overexpressing cells include *Rpa1 Topbp1, H2afx, Xrcc2*, and *Blm*. RPA1 is a protein that binds to resected DNA during HRR to avoid the formation of secondary structures that can obstruct RAD51 recombinase [54]. It has been shown that reducing RPA1 expression can reduce DSB repair, alter DNA replication, and increase the sensitivity of cells to IR [55,56]. TOPBP1 is a protein that functions in DNA damage signaling and mediates RAD51 phosphorylation and activation to replace RPA1 during HRR [57]. The *H2afx* gene encodes the H2A histone variant H2AX, which plays a crucial role in DSB signaling. DSBs are signaled by H2AX S139 phosphorylation by ATM, ATR, or PRKDC (DNA-PKcs) [1]. Therefore, H2AX dysregulation can have a negative impact on the repair capacity of DSBs by compromising their correct signaling. XRCC2 is a RAD51 paralog known to act as a mediator in the replacement of RPA1 by RAD51 during HRR [58]. The decrease in the expression of XRCC2 has been associated with a delay in the repair of DSBs [59]. BLM is a helicase involved in DNA replication and repair. During HRR, BLM contributes to DNA resection to generate the single-stranded section of DNA to which RPA1 then binds and has also been shown to regulate RAD51-mediated recombination [60].

We also observed the downregulation of other genes associated with DSB repair, DSB signaling, and cell cycle regulation. These genes include *Abl1, Cdc25c, Fanca, Fancg, Mcph1, Mlh3, Ppp1r15a, Pttg1,* and *Rnf8* (Table 2). ABL1 can phosphorylate proteins involved in DSB repair, such as DNA-PKcs, RAD51, RAD52, BRCA1, RAD9, and TOPBP1 [61,62,63,64,65,66,67], thus indicating that ABL1 plays a relevant role in the regulation of DSB repair and cell cycle arrest. CDC25C is phosphorylated and inactivated by CHK1/CHK2 in an ATM-dependent manner to arrest the cell cycle in G2 to allow DNA damage repair [68]. MCPH1 has been shown to mediate ATM and ATR signaling during DNA damage response [69]. MCPH1 downregulation has been associated with decreased MDC1–53BP1–ATM foci formation, necessary for the repair of IR-generated DSBs [70]. It has also been reported that MCPH1, when activated by ATM, interacts with TOPBP1, which, in turn, activates ATR to amplify replicative stress signaling [71]. MCPH1 also interacts with BRCA2 and RAD51 during HRR and participates in the modulation of the G2-M checkpoint by regulating the expression of BRCA1 and CHK1 [72,73]. *Ppp1r15a* (*Gadd34*) is associated with cell cycle arrest, and its expression is induced in response to DNA damage. Increased *Ppp1r15a* expression has been associated with increased apoptosis of cells with DNA damage [74]. PTTG is involved in sister chromatid separation during mitosis [75]. Additionally, increased expression of *Pttg1* causes errors in chromosome segregation during mitosis due to increased genomic instability [76]. RNF8 functions in the signaling of DSBs through the mono-ubiquitination of H2A and H2B [77,78]. RNF8 deficiency has been associated with tumor development, probably due to increased genomic instability [79]. FANCA and FANCG (XRCC9) interact with each other and participate in the repair of interstrand crosslinks and DSBs via the Fanconi anemia pathway. FANCA and FANCG interact with FANCB, FANCC, FANCE, FANCF, FANCL, and FANCM to form the FA complex. The FA complex monoubiquitinates FANCD2 and FANCI proteins, which, in turn, colocalize with BRCA1, BRCA2, and RAD51 at the sites of damage [80]. It has been seen that FANCA acts like RAD52, regulating strand alignment and exchange during DSB repair by HRR; such activity is stimulated by interaction with FANCG [81]. Dysregulation of the aforementioned genes, thus, negatively affects the DNA damage signaling and cell cycle regulation necessary for a correct DNA repair. This suggests that the reduction in DNA repair capacity in cells that overexpress miR-27b-3p is not only due to negative alterations in HRR and BER but also in the signaling of DNA damage, particularly DSBs, which prevent the adequate cell cycle control needed for proper DNA repair.

The results obtained through the analysis of expression patterns and Reactome of the negative regulatory role of miR-27b-3p on BER, HRR, and cell cycle genes assume the dysfunction of reparative capacity (Figure 2B and Figure 3; Table 2). However, the loss of reparative capacity due to overexpression of miR-27b-3p was evident through the induction of damage by DOX. For this reason, when comparing the expression patterns between BALB/c-3T3 cells treated with DOX and those that overexpress miR-27b-3p treated with DOX (Figure 4), it was shown that the genes of the Fanconi anemia pathway and HRR are involved in the loss of function of damage repair (Figure 1I,J and Figure 5). This result is not surprising since it is known that the canonical function of FA proteins is to collaborate with several other DNA repair proteins (HRR, TLS) to eliminate the clastogenic effects of DNA interstrand crosslinks (ICLs). Recent discoveries have revealed that the FA pathway functions in a critical tumor suppressor network to preserve genomic integrity by stabilizing replication forks, mitigating replication stress, and regulating cytokinesis [82].

The functioning of miR-27b-3p in cancer, as occurs with other miRNAs, will depend on specific contextual determinants, including intrinsic and extrinsic cellular features of tumors and target mRNAs [83,84]. The results of the present study indicate that miR-27b-3p functions as a negative regulator of DNA repair. When this miRNA is overexpressed, it can deregulate the expression of genes involved in DNA repair mechanisms, mainly HRR, the Fanconi anemia pathway, and BER, as well as other genes that participate in DSB signaling and cell cycle regulation. It should be noted that the interpretation derived from the present study should be corroborated by studies that compare the behavior of cancer cells of the cervix, breast, and glioma (where miR-27b-3p is overexpressed) with the behavior of cancer cells of the prostate, colorectal, gastric, bladder and lung (where miR-27b-3p is underexpressed) [26,27,28,29,30].

Under the genotoxic stress of doxorubicin, we were able to dissect a pattern of genes that explain the loss of reparative function that we observed with the comet assay. Protein interaction analysis indicates that this “genic signature” (*Dclre1a, Rev1, Xpa, Pms2, Brca2, Parp2, Smc3, Nbn, Bax*) is mainly involved in the Fanconi anemia pathway and homologous recombination repair. This acquires relevance since the Fanconi anemia pathway, being strongly related to the acquisition of chemoresistance in cancer treatment, opens the possibility of proposing an adjuvant treatment through the overexpression of miR-27b-3p and DOX (or some drug that acts similarly) to favor the downregulation of the nine genes obtained from the protein interaction analysis. Additionally, the study of the intrinsic and extrinsic factors that determine the expression of miR-27b-3p and the regulation of its target genes will allow us to delineate the importance of this miRNA in the treatment of cancer.

## Figures and Tables

**Table 1:** Types of DNA lesions induced by the xenobiotic agents used and the DNA repair mechanisms that remove them.

DNA Damaging Agent and Employed Dose/Concentration	Main Type of DNA Lesion Induced	Associated DNA Repair Mechanism (Alternative)
UV (126 mJ)	CPDs, 6-4PPs	NER, (TLS)
FeCl_3_ (100 µM)	Oxidative lesions through ROS generation	BER, (TLS)
Ionizing radiation (7.5 Gy)	DSBs, SSBs, oxidative lesions	NHEJ, HRR, BER, (TLS)
Doxorubicin (50 µM)	DSBs, oxidative lesions	NHEJ, HRR, BER, (TLS)

CPD: cyclobutane–pyrimidine dimers; ROS: reactive oxygen species; DSBs: double-strand breaks; SSBs: single-strand breaks; NER: nucleotide excision repair; TLS: translesion synthesis; BER: base excision repair; NHEJ: nonhomologous end joining; HRR: homologous recombination repair.

**Table 2:** Most enriched processes by deregulated genes in cells that overexpress miR-27b-3p.

Pathway Name	*p*-Value	FDR	Associated Genes
Homology Directed Repair	1.11 × 10^−16^	6.66 × 10^−15^	*Blm; Fen1; Rad52; Pcna; H2afx; Rpa1; Rnf8; Hus1; Topbp1; Rad9a; Xrcc2; Abl1; Xrcc1; Nbn; Pole*
HDR through Homologous Recombination (HRR) or Single-Strand Annealing (SSA)	1.11 × 10^−16^	6.66 × 10^−15^	*Blm; Rad52; Pcna; H2afx; Rpa1; Rnf8; Hus1; Topbp1; Rad9a; Xrcc2; Abl1; Nbn; Pole*
DNA Double-Strand Break Repair	1.11 × 10^−16^	6.66 × 10^−15^	*Blm; Fen1; Rad52; Pcna; H2afx; Rpa1; Rnf8; Hus1; Topbp1; Rad9a; Xrcc2; Abl1; Xrcc1; Nbn; Pole*
DNA Repair	1.11 × 10^−16^	6.66 × 10^−15^	*Blm; Fen1; Rad52; Pcna; Mgmt; Mpg; H2afx; Apex1; Rpa1; Rnf8; Xpa; Hus1; Topbp1; Rad9a; Ung; Xrcc2; Abl1; Fanca; Xrcc1; Nbn; Pole; Fancg*
HDR through Single-Strand Annealing (SSA)	5.36 × 10^−14^	2.57 × 10^−12^	*Blm; Rad52; Rpa1; Hus1; Abl1; Topbp1; Nbn; Rad9a*
HDR through Homologous Recombination (HRR)	1.01 × 10^−13^	4.04 × 10^−12^	*Blm; Pcna; Xrcc2; Rpa1; Hus1; Topbp1; Nbn; Rad9a; Pole*
Cell Cycle	1.39 × 10^−13^	4.73 × 10^−12^	*Blm; Pcna; Fen1; Pttg1; H2afx; Rpa1; Rnf8; Mcph1; Hus1; Topbp1; Rad9a; Mlh3; Abl1; Nbn; Cdc25c; Pole*
Resolution of Abasic Sites (AP sites)	2.78 × 10^−13^	7.47 × 10^−12^	*Pcna; Fen1; Mpg; Apex1; Rpa1; Xrcc1; Pole; Ung*
G2/M DNA Damage Checkpoint	2.87 × 10^−13^	7.47 × 10^−12^	*Blm; H2afx; Rpa1; Rnf8; Hus1; Topbp1; Nbn; Rad9a; Cdc25c*
Base Excision Repair	1.7 × 10^−12^	4.08 × 10^−11^	*Pcna; Fen1; Mpg; Apex1; H2afx; Rpa1; Xrcc1; Pole; Ung*

## Supplementary Materials

The following are available online at https://www.mdpi.com/article/10.3390/genes12091333/s1, Table S1: Representative cellular processes by genes deregulated by miR-27b-3p; Figure S1: Representative comet images.
