# Supplementary material for: miR-27b-3p a Negative Regulator of DSB-DNA Repair
Source: Genes (Basel). 2021 Aug 27;12(9):1333. doi: 10.3390/genes12091333 (PMC8471791; doi:10.3390/genes12091333)
Supplement: Supplementary file 1 [file genes-12-01333-s001.zip › suplementary material/Table S1.pdf]

Table S1. Cell survival after several genotoxic agents

	Non-transfected		miR-27b-3p mimic		anti-miR-27b-3p	
**Treatment**	**Mean**	**SD**	**Mean**	**SD**	**Mean**	**SD**
Control	82.4	6.58	82.2	7.56	81	5.61
UV	75	3.67	76.8	3.11	77.4	4.51
$FeCl_3$	88	4.85	83.4	4.34	83.6	6.47
IR	77.4	5.81	79.2	2.17	78.6	7.02
DOX	78	5.24	77.2	4.87	79.8	3.27

Data represents the mean percentages and standard deviations (SD) of five independent measurements. According to Student's t- test, no statistical differences were found between control and each genotoxic agents in non-transfected cells and under miR-27b-3p mimic or anti-miR-27b-3p conditions.
