# Supplementary figures and images for: miR-27b-3p a Negative Regulator of DSB-DNA Repair
Source: Genes (Basel). 2021 Aug 27;12(9):1333. doi: 10.3390/genes12091333 (PMC8471791; doi:10.3390/genes12091333)

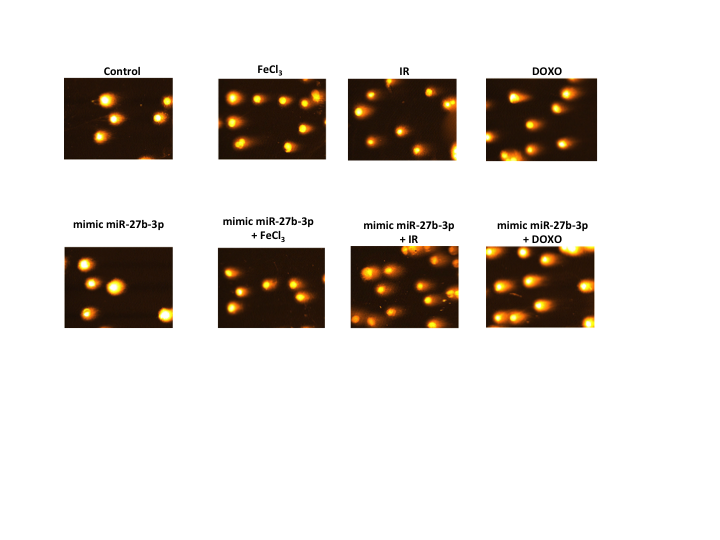

Supplement: Supplementary file 1 [file genes-12-01333-s001.zip › suplementary material/Figure S1.tiff]
